# Shexiang Baoxin Pill Corrects Metabolic Disorders in a Rat Model of Metabolic Syndrome by Targeting Mitochondria

**DOI:** 10.3389/fphar.2018.00137

**Published:** 2018-03-02

**Authors:** Dan Wei, Ningning Zheng, Lanyan Zheng, Leting Wang, Liang Song, Luning Sun

**Affiliations:** ^1^Department of Pathophysiology, College of Basic Medical Science, China Medical University, Shenyang, China; ^2^Department of Pathogen Biology, College of Basic Medical Science, China Medical University, Shenyang, China; ^3^Shanghai Hutchison Pharmaceuticals, Shanghai, China; ^4^Wanleibio Co., Ltd., Shenyang, China

**Keywords:** metabolic syndrome, Shexiang Baoxin Pill, lipid metabolism, inflammation, mitochondrial proteins, reactive oxygen species

## Abstract

**Background:** Metabolic syndrome (MS) is a global epidemic that has great socioeconomic and public health implications. This study reports observed effects of the Shexiang Baoxin Pill (SBP) in a rat model of MS and explores its underlying mechanisms of action.

**Methods:** A diet-induced rat model of MS was established according to accepted methods, and the rats were randomly divided into two groups: a control group (0.9% NaCl, 100 mg/kg^•^d) and a SBP-treated group (SBP, 100 mg/kg^•^d). Systolic blood pressures, fasting blood glucose (FBS) levels, triglyceride (TG) levels, high-density lipoprotein cholesterol (HDL-C) levels, body weights, and abdominal perimeters were dynamically monitored and analyzed. Serum leptin, adiponectin, TNF-α, IL-6, and IL-10 levels were measured by ELISA. Leptin, adiponectin, TNF-α, IL-6, and IL-10 expression in adipose tissue, as well as AMP-activated protein kinase (AMPK) and peroxisome proliferator-activated receptor gamma coactivator 1-α (PGC-1α) expression in heart, liver, skeletal muscle, and adipose tissue was measured by western blot. Expression of the mitochondrial protein UCP2, Cytochrome b and ATPase was observed by immunofluorescent staining.

**Results:** SBP significantly decreased serum TG, TC, LDL-C levels and increased HDL-C levels. SBP also optimized the leptin/adiponectin ratio by decreasing leptin expression and increasing adiponectin expression in adipose tissue. SBP antagonized inflammatory reactions by promoting IL-10 expression in adipose tissue while inhibiting TNF-α and IL-6 expression. SBP improved lipid metabolism by up-regulating the expression of AMPK and PGC-1α. Furthermore, SBP decreased the severity of MS and its complications by adjusting the expression of several mitochondrial proteins, including UCP2, Cytochrome b and ATPase.

**Conclusion:** SBP exhibits prominent therapeutic effects in the setting of MS. Possible mechanisms of action may be related to its anti-inflammatory and anti-oxidative characteristics, as well as its effects on improving lipid metabolism and protecting mitochondrial function.

## Introduction

The incidence of metabolic syndrome (MS) is on the rise globally. Approximately one–fourth of adults worldwide were reported to have been diagnosed with MS (Kassi et al., 2011). Nearly 35% of all adults and 50% of those aged 60 years or older were reported to suffer from MS in the United States (Aguilar et al., 2015). A recent meta-analysis concluded that the pooled estimate of MS prevalence among subjects was 24.5% in Mainland China (Li et al., 2016). MS encompasses a cluster of risk factors for cardiovascular disease (CVD) including abdominal obesity, dyslipidemia, hypertension, and hyperglycemia (Ford, 2005). As a result, patients with MS are at twice the risk of developing CVD and have 1.80 and 2.05 times higher statistically significant probabilities for myocardial infarct (MI) and stroke events, respectively, compared to individuals without the syndrome (Alberti et al., 2009; Kazlauskiene et al., 2015). Moreover, the risk of developing type 2 diabetes mellitus (DM) was reported to be five times as great in MS patients when compared to those without the syndrome (Alberti et al., 2009). MS patient mortality due to cardiovascular complications is understandably significantly increased as well. As there is no single, specific treatment for MS at present, lowering body weight, blood pressure, blood lipid levels, and improving insulin resistance are the only generally accepted management options available. However, long-term use of multi-drug regimens will inevitably lead to undesired side effects and mutual antagonism. Therefore, an effective medication with minimal side effects is urgently required.

Shexiang Baoxin Pill (SBP) is a traditional Chinese medicine (TCM) formulation comprised of seven substances common to the TCM field, including Moschus, Radix Ginseng, Calculus Bovis, Styrax, Cortex Cinnamomi, Venenum Bufonis, and Borneolum Syntheticum (**Table 1**). It originates from the ancient Suhexiang Pill prescription recorded in *Prescriptions of the Bureau of Taiping People’s Welfare Pharmacy*, at one time a popular medication for chest pain or discomfort caused by coronary heart disease (CHD) in China (Liu et al., 2015; Cen et al., 2017; Fang et al., 2017; Zhang et al., 2017). In recent years, greater attention has been paid to its roles in modulating cellular metabolism. Among the 40 plus volatile and 70 non-volatile chemical species identified in SBP, 2 bile acids, ursodeoxycholic acid and chenodeoxycholic acid have been approved by the U.S. Food and Drug Administration (FDA) and marketed as ursodiol and chenodiol, respectively. These compounds can inhibit the production of cholesterol, and thereby treat hypertriglyceridemia (Watanabe et al., 2004; Liu et al., 2015). SBP has been reported to relieve spontaneous hypertension, protect pancreatic β-cells, improve insulin resistance and enhance the activity of insulin (Jinqin et al., 2011). In addition, SBP has also been reported to attenuate mitochondrial injury of myocardiocytes in MS rat models (Zhang et al., 2008; Liu et al., 2012).

**Table 1:** Composition of Shexiang Baoxin Pill (SBP).

Chinese medical materials	Compounds	Effects (Actions)
Moschus	Muscone	Induces resuscitation and refreshes the mind, activates blood and alleviates pain, anti-inflammatory.
Radix ginseng	Ginsenoside Rb1 Ginsenoside Re Ginsenoside Rb2	Induces tranquilization of the mind and improves intelligence, nourishes the blood, improves glucose and lipid metabolism. Exhibits anti-atherosclerotic and cardiotonic effects.
Calculus bovis	Cholic acid Ursodeoxycholic acid Chenodeoxycholic acid Deoxycholic acid	Extinguishes wind and stops spasms, clears phlegm and thereby eases resuscitation, clears heat and relieves toxicity. Decreases cholesterol and triglyceride levels, exhibits anti-inflammatory and cardiotonic effects.
Styrax	Benzyl benzoate	Induces resuscitation and refreshes the mind, dispels cold and alleviates pain. Exhibits anti-inflammatory and anti-thrombotic properties.
Cortex Cinnamomi	Cinnamic acid Cinnamaldehyde	Dispels cold and stops pain, warms meridians and collaterals, and exhibits anti-hypertensive and anti-diabetic properties.
Venenum Bufonis	Bufalin Cinobufagin Gamabufotalin	Induces resuscitation, relieves toxicity and alleviates pain, and exhibits anti-inflammatory, anti-coagulative and cardiotonic properties.
Borneolum	Borneol	Induces resuscitation and refreshes the mind, clears heat and alleviates pain. Exhibits anti-inflammatory and anti-hypoxic properties.


This study reports the therapeutic effects exerted by SBP on MS in a diet-induced MS rat model and explores the underlying mechanisms of its action, particularly focusing on the center of cellular metabolism, the mitochondrion.

## Materials and Methods

### Materials

A batch of SBP (batch number 130401) was kindly donated by the Shanghai Hutchison Pharmaceuticals Company (Shanghai, China).

### Animals

Adult male Sprague-Dawley (SD) rats (weight 210 g ± 20 g) were housed under a standard light-dark cycle and provided free access to food and water. Animal facilities were used with the permission of the Institutional Animal Care and Use Committee of China Medical University. Animal care protocols adhered to were approved by this committee as well.

An MS rat model was established by feeding a high-fat, high-sugar and high-salt diet (20% crude fat, 10% sugar, 5% salt, 0.5% bile salt, 2% cholesterol, 52.5% standard feed) to rats for 16 weeks. A control group was fed standard feed. After fasting the animals for 12 h overnight, 1.0 ml of blood was collected from each rat’s orbital vein and centrifuged at 3,000 *g* for 15 min to obtain serum. Body weights, abdominal perimeters, and levels of fasting blood glucose (FBS), triglycerides (TG), total cholesterol (TC), low density lipoprotein-cholesterol (LDL-C), and high density lipoprotein-cholesterol (HDL-C) were measured every 4 weeks. Systolic blood pressures were measured using the tail-cuff method after anesthetizing rats by inhalation of diethyl ether: three readings were taken consecutively with an average calculated and considered as a final systolic blood pressure value. 24 SD rats meeting MS criteria (Aleixandre de Artinano and Miguel Castro, 2009) were confirmed after 16 weeks.

### Administration of SBP

Metabolic syndrome rats were randomly divided into one control group (*n* = 12) and one SBP treatment group (*n* = 12). According to guidelines of clinical SBP dosage (2.25 mg/kg/d), 20 mg/kg/d SBP was administered intragastrically for 12 weeks. A corresponding volume of saline solution was administered to rats in the control group. The high-fat, high-sugar and high-salt diet was fed to both groups throughout those 12 weeks. After a 12 h overnight fast following final administrations, the concentrations of serum TC, TG, LDL-C, HDL-C, and FBG were measured.

### Enzyme-Linked Immunosorbent Assay

Serum TNF-α, IL-6, IL-10, leptin and adiponectin levels were measured in duplicate by ELISA (adiponectin, TNF-α, IL-6 and IL-10 ELISA kit: R&D Systems, Minneapolis, MN, United States; leptin ELISA kit: Abcam, Cambridge, United Kingdom) according to the manufacturer’s instructions.

### Tissue Processing

Animals were anesthetized with 10% chloral hydrate (300 mg/kg, i.p.). Hearts, livers, abdominal adipose tissue and gastrocnemius muscles were rapidly excised and thoroughly rinsed after rat decapitation. All tissues were dissected into two portions (with one stored at -80°C as a backup for Western blot analysis) and the remains were fixed in 10% neutral buffered formalin for 3 days and embedded in paraffin. Embedded tissues were sectioned into 5-μm slices and processed for immunofluorescent staining.

### Western Blot Assay

Protein lysates were subjected to 12% SDS-PAGE and transferred to polyvinylidene fluoride membranes, respectively. After blocking with 5% skimmed milk for 2 h, membranes were incubated overnight at 4°C along with their respective primary polyclonal antibodies [adiponectin 1:500, leptin 1:1000, AMP-activated protein kinase (AMPK) 1:1000, PGC-1α 1:200, Abcam, United States; TNFα1:1000, IL-6 1:500, IL-10 1:1000, Abnova, Taiwan]. The membranes were then incubated with appropriate secondary horseradish peroxidase-conjugated anti-rabbit or anti-goat immunoglobulin G (IgG) antibodies at a 1:5000 dilution for 45 min at 37°C. Blots were visualized using an enhanced chemoluminescence (ECL) reagent (Beyotime, China) and quantified by scanning densitometry.

### Immunofluorescent Staining

Dehydrated and dewaxed sections were washed in PBS and epitopes were retrieved in antigen retrieval solution for 10 min at 100°C. Following washing with PBS, sections were incubated at room temperature for 1 h with 0.01 M PBS containing 5% NGS and 0.3% Triton X-100 to inhibit non-specific staining. Subsequently, sections were treated with primary antibodies (UCP2 1:50, Cytochrome b 1:50, Santa, United States; ATPase 1:100, Biorbyt, United States) in the blocking buffer at 4°C overnight. After thoroughly washing the sections, they were incubated with the appropriate secondary Cy 3-labeled anti-rabbit or anti-mouse antibodies in 0.01 M PBS at a 1:100 dilution at room temperature for 1 h in the dark. Sections were then washed three times in 0.01 M PBS and counterstained with a 1 μg/μL 4′, 6-diamidino-2-phenylindole (DAPI, Biosharp, China) solution before being mounted on slides using PermaFluor (Thermo Fisher Scientific). Stained samples were analyzed with a fluorescence microscope (BX51, Olympus Corporation).

### Statistical Analysis

All data were analyzed using the unpaired two-sample Student’s *t*-test. Data are expressed as mean ± SE (Standard Error) and were deemed statistically significant when *P* < 0.05.

## Results

### SBP Improved Blood Glucose and Lipid Levels of MS Rats

**FIGURE 1:**
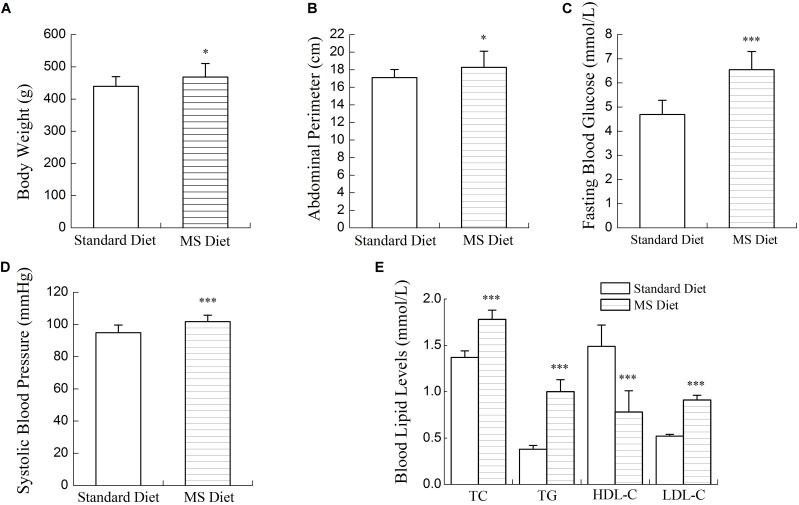
Physiological indicators of the diet-induced metabolic syndrome (MS) rat model. Body weights **(A)**, abdominal perimeters **(B)**, systolic blood pressures **(C)**, and blood glucose levels **(D)** in MS rats increased significantly compared to normal controls. Blood lipid levels **(E)** of MS rats were abnormal; TC, TG, LDL-C increased and HDL-C decreased significantly compared to normal controls. Values are expressed as mean ± SE. ^∗^*P* < 0.05, ^∗∗∗^*P* < 0.001 vs. standard diet.

**FIGURE 2:**
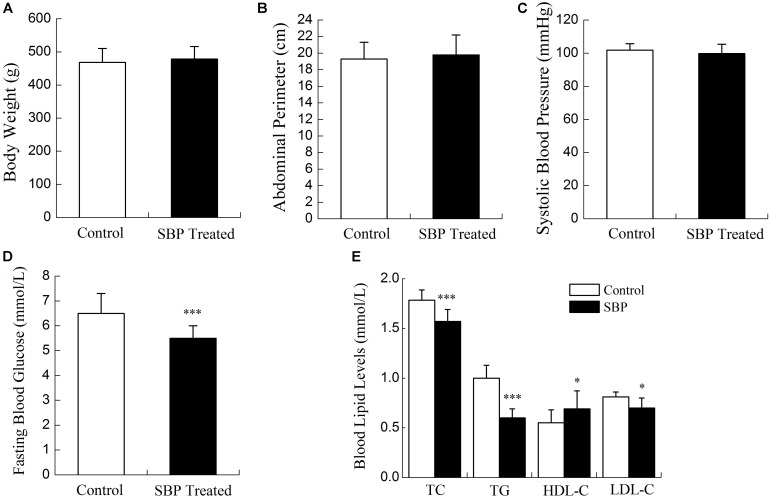
Physiological indicators of the MS rat model after treatment with SBP. Body weights **(A)**, abdominal perimeters **(B)**, and systolic blood pressures **(C)** of MS rats decreased slightly after treatment with Shexiang Baoxin Pill (SBP). These readings were not considered to be statistically significant. FBG **(D)** decreased after 12 weeks of SBP administration. TC, TG, and LDL-C decreased and HDL-C **(E)** increased after 12 weeks of SBP administration. Values are expressed as mean ± SE. *n* = 12 for each group. ^∗^*P* < 0.05, ^∗∗∗^*P* < 0.001 vs. control group.

Compared with rats fed the standard diet, MS rats had increased body weights, abdominal perimeters and systolic blood pressures. Blood lipid and glucose levels were abnormal in MS rats. As shown in **Figures 1**, **2**, after 12 weeks’ SBP therapy, concentrations of serum TC, TG, LDL-C, and FBG decreased, while the concentration of HDL-C increased in comparison to the control group. Body weights, abdominal perimeters and systolic blood pressures also decreased slightly; however, no statistically significant differences between SBP treated and control groups were noted.

### SBP Regulated the Secretion of Leptin and Adiponectin

**FIGURE 3:**
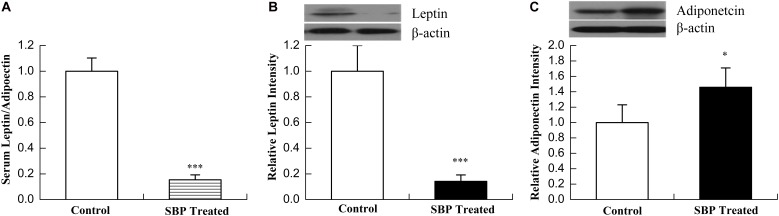
Shexiang Baoxin Pill regulated the secretion of leptin and adiponectin. Ratio of serum leptin/adiponectin **(A)**. SBP optimized the ratio of serum leptin/adiponectin in MS rats by decreasing levels of serum leptin and increasing levels serum adiponectin. Expression of leptin **(B)** decreased while expression of adiponectin **(C)** increased in adipose tissue of MS rats after treatment with SBP. Values are expressed as mean ± SE. *n* = 12 for **(A)**; *n* = 3 for **(B,C)**. ^∗^*P* < 0.05, ^∗∗∗^*P* < 0.001 vs. control group.

Leptin and adiponectin are both adipokines produced mainly by adipose tissue, and are involved in the regulation of various biological processes such as glucose and lipid metabolism, energy expenditure and inflammatory responses. A leptin/adiponectin imbalance was found to be associated with increased waist circumference, a diminished vascular response to acetylcholine and greater vasoconstriction in response to angiotensin II (Lopez-Jaramillo, 2016). In the present study, we examined serum concentrations of leptin and adiponectin by ELISA. Subsequently, their expression levels in adipose tissue were measured by Western blot. As shown in **Figure 3**, the concentration of serum leptin decreased while that of adiponectin increased after SBP treatment, compatible with their expression in adipose tissue.

### SBP Inhibited the Inflammatory Reactions

**FIGURE 4:**
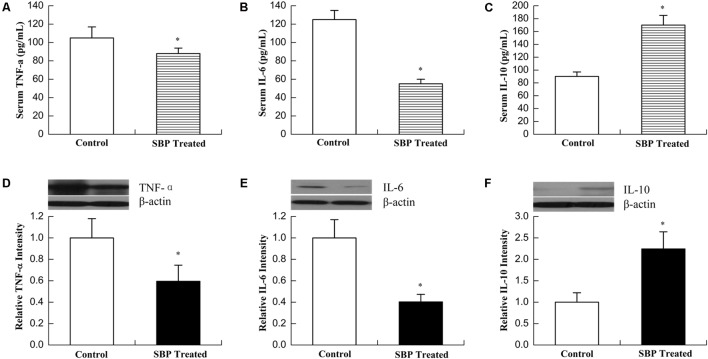
Shexiang Baoxin Pill inhibited inflammatory reactions. Serum concentrations of TNFα **(A)**, IL-6 **(B)**, and IL-10 **(C)**. Levels of the proinflammatory cytokines TNFα and IL-6 decreased while levels of the anti-inflammatory cytokine IL-10 increased after treatment with SBP. Expression of TNFα **(D)**, IL-6 **(E)**, and IL-10 **(F)** in adipose tissue. The expression of TNFα and IL-6 decreased while that of IL-10 increased in adipose tissue of MS rats after treatment with SBP. Values are expressed as mean ± SE. *n* = 12 for **(A–C)**; *n* = 3 for **(D–F)**. ^∗^*P* < 0.05, ^∗∗∗^*P* < 0.001 vs. control group.

Metabolic syndrome results in an imbalance between energy intake and expenditure, also typically associated with the presence of low-grade chronic inflammation and abnormal cytokine production. This persistent inflammatory state, co-existing with insulin resistance, is associated with a variety of adverse outcomes, including DM and CVD (Piantedosi et al., 2016). As shown in **Figure 4**, the serum concentration of the proinflammatory cytokines TNFα and IL-6 decreased while that of the anti-inflammatory cytokine IL-10 increased after treatment with SBP, compatible with their expression levels in adipose tissue.

### SBP Regulated the Expression of AMPK and PGC-1α in Different Tissues

**FIGURE 5:**
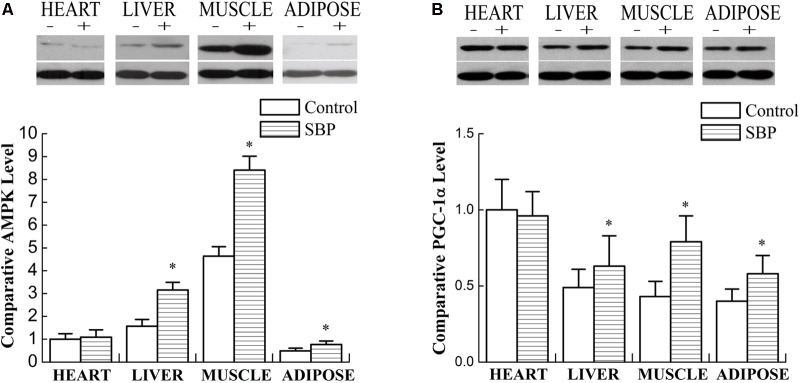
Shexiang Baoxin Pill regulated the expression of AMPK and PGC-1α in different tissues. **(A)** Western blotting of AMPK in heart, liver, skeletal muscle, and adipose tissue. The expression of AMPK increased markedly in liver and skeletal muscle and increased slightly in adipose tissue after treatment with SBP. **(B)** Western blotting of PGC-1α in heart, liver, skeletal muscle, and adipose tissue. The expression of PGC-1α increased markedly in liver, skeletal muscle, and adipose tissue after treatment with SBP. Values are expressed as mean ± SE, *n* = 3 for each group. ^∗^*P* < 0.05 vs. control.

AMP-activated protein kinase is a key modulator of lipid metabolism and energy balance. Its activation stimulates ATP-generating metabolic pathways and inhibits anabolic reactions in liver and skeletal muscle tissue. Thus, AMPK signaling is crucial for the regulation of fatty acid oxidation, mitochondrial biogenesis, glucose uptake and the control of insulin sensitivity in skeletal muscle (Tan et al., 2012). PGC-1α is a key nuclear receptor co-activator vital in fatty acid oxidation that can also induce mitochondrial biogenesis. An increase in PGC-1α expression can promote oxidation of long-chain fatty acids in adipocytes (Tan et al., 2012). As shown in **Figure 5**, the expression of AMPK and PGC-1α increased remarkably in liver and skeletal muscle tissue after treatment with SBP. Additionally, the expression of PGC-1α also increased in adipose tissue. However, this effect was not observed in myocardiocytes.

### SBP Regulated the Expression of Several Mitochondrial Membrane Proteins in Different Tissues

**FIGURE 6:**
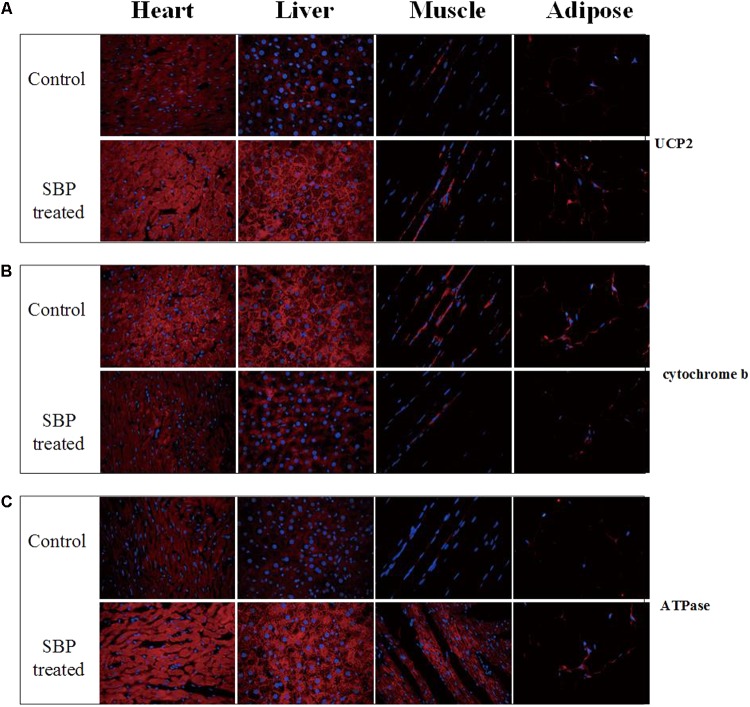
Shexiang Baoxin Pill regulated expression of mitochondrial proteins UCP2, cytochrome b and ATPase in different tissues. **(A)** Expression of UCP2 increased in different tissues of MS rats, especially in cardiomyocytes, after treatment with SBP (merged, 600×). **(B)** Expression of cytochrome b decreased in different tissues of MS rats after treatment with SBP (merged, 600×). **(C)** Expression of ATPase increased in different tissues of MS rats after treatment with SBP (merged, 600×).

The mitochondrion produces energy and maintains an intracellular redox status. Moreover, it is also an organelle where most reactive oxygen species (ROS) are produced. In the present study, the expression of several mitochondrial membrane proteins in different tissues was detected by immunofluorescent staining. As shown in **Figure 6**, expression of uncoupling protein 2 (UCP2), a mitochondrial protein that can dissipate mitochondrial *trans*-membrane potential, increased in several tissues of MS rats, especially in the heart, after SBP treatment. At the same time, the expression of cytochrome b, the main subunit of mitochondrial complex III, was down-regulated in the liver, heart, skeletal muscle, and adipose tissues of MS rats. However, dissipation of the mitochondrial *trans*-membrane electrochemical gradient might result in decreased ATP production, an unfavorable biological adaptation. As expected, we found the expression of ATPase (mitochondrial complex V) to be up-regulated after treatment with SBP for 12 weeks.

## Discussion

Insulin resistance, central obesity and disorders of adipose tissue were thought to be the etiologic factors of MS, with heredity, aging, lack of physical activity and the inflammatory response also contributing to the pathogenesis of this condition (Okosun et al., 2000). To date, the accepted pharmacological management of MS has been the long-term use of multi-drug regimens targeting multiple pathologic processes; however, clinical outcomes remain subpar due to multiple adverse effects and poor efficacy. An ideal candidate compound would be safe, effective, and simultaneously targeting multiple pathologic mechanisms of MS. Not only has SBP been previously used to treat heart disease, it can also attenuate the general manifestations of MS including spontaneous hypertension, hypertriglyceridemia and insulin resistance. Importantly, SBP can diminish mitochondrial injury and improve cellular metabolism. All such findings suggest that SBP has great promise in effectively treating MS.

In the present study, we found that SBP could remarkably alleviate the major manifestations of metabolic disorders in the MS rat model. To further clarify the therapeutic mechanisms, first, we observed the effects of SBP on the production of adipokines, including leptin and adiponectin. Abdominal obesity with increased visceral fat is one of the major components of MS. At present, adipose tissue has been defined as an endocrine organ, which can produce adipokines to regulate various processes, such as glucose and fatty-acid metabolism, the inflammatory response and cardiovascular function (Sartori et al., 2016). Adiponectin is a 30 kDa secretory protein that is produced mainly by adipocytes, and its main functions consists of the upregulation of insulin and maintenance of energy homeostasis (Lihn et al., 2005). Adiponectin functions as an insulin-sensitizing hormone which can reduce hepatic glucose production and enhance hepatic insulin-mediated processes. Functional and genetic studies have suggested that a reduction in adiponectin levels played a major role in the development of insulin resistance (IR), DM, atherosclerosis and MS (Yamauchi et al., 2001). Usually, plasma adiponectin levels decrease in obesity. Leptin is the 167-amino acid product of the human leptin gene (ob gene), and is primarily secreted by white adipose tissue. The functions of leptin include mediation of food intake, liver glucose production, suppression of lipogenesis in adipose tissue, and modulation of the immune response (Ewart-Toland et al., 1999). Chronically elevated leptin levels in obesity may result in decreased responsiveness of pancreatic β-cell receptors and lead to increased insulin secretion, contributing to the occurrence of insulin resistance (Thorand et al., 2010). In MS, increased visceral adipose tissue disturbs adipokine secretion and leads to a chronic state of low-grade inflammation mediated by the infiltration of macrophages into adipose tissue (Galic et al., 2010). Although leptin and adiponectin were considered important components of MS, the leptin/adiponectin (L/A) ratio plays a greater role than either one of these hormones alone (Oda et al., 2008). Leptin and adiponectin have opposite effects on subclinical inflammation. While leptin upregulates proinflammatory cytokines such as TNF-α and IL-6, adiponectin exhibits anti-inflammatory properties and downregulates the expression of a number of proinflammatory immune mediators. Therefore, an L/A imbalance promotes development of a proinflammatory state and hence a progression of MS. Our study revealed that SBP stimulated adipose tissue to secret adiponectin, but inhibited the secretion of leptin. As a result, the L/A ratio greatly decreased. Serum concentrations of proinflammatory cytokines TNFα and IL-6 correspondingly decreased while concentrations of the anti-inflammatory cytokine IL-10 increased after treatment with SBP, consistent with their expression levels in adipose tissue. The aforementioned findings revealed that SBP optimized the L/A ratio and created an anti-inflammatory state, improving metabolism.

Adiponectin, secreted from adipose tissue, exerts its anti-diabetic effects via activation of AMPK and peroxisome proliferator-activated receptor (PPAR) pathways in insulin-sensitive organs, importantly liver and skeletal muscle tissue, which are the principal storage sites of glucose and fatty acids (Okada-Iwabu et al., 2013). AMPK is a key regulator of metabolic homeostasis expressed ubiquitously in eukaryotic cells (Hardie et al., 2016). It has been reported that the anti-diabetic drug metformin could increase AMPK phosphorylation and ultimately improve glucose metabolism (Zhang et al., 2009). Of note, AMPK activation can induce post-translational phosphorylation of PGC 1α. As our results revealed, the expression of AMPK and PGC-1α increased substantially in liver and skeletal muscle tissue after treatment with SBP, suggesting that SBP can attenuate insulin resistance via the AMPK-PGC-1α pathway. AMPK and PGC-1α also regulate mitochondrial biogenesis, with PGC-1α in particular playing a major regulatory role in this process (Puigserver et al., 1998; Lin et al., 2002). AMPK, as an energy-sensitive kinase, phosphorylates PGC-1α on threonine-177 and serine-538, thereby stimulating its activation (Jager et al., 2007). Mitochondria utilize the electron transport chain, or oxidative phosphorylation, within their inner membrane to produce ATP. In mammalian mitochondria, the oxidative phosphorylation system is mainly formed by five complexes. Complex III produces superoxide on both sides of the inner mitochondrial membrane and is therefore the most important site in the electron transport chain, while uncoupling proteins (UCPs) are specific mitochondrial membrane proteins suggested to exert protective effects on mitochondria by dissipating *trans*-membrane potential and decreasing complex III ROS generation. In the present study, we found that the expression of the protein UCP2 increased in a variety of tissues, especially in the heart, after treatment with SBP. At the same time, expression of cytochrome b, the main subunit of the mitochondrial complex III, appeared to be down-regulated by SBP. Our findings indicate that SBP could in particular exert cardioprotective effects in the MS rat model by reducing production of ROS. Interestingly, to avoid an energy deficiency that could arise from dissipation of the mitochondrial *trans*-membrane electrochemical gradient, SBP up-regulated the expression of ATPase as a compensatory mechanism.

Although systolic blood pressure also decreased slightly in the animals studied, there was no statistical significance between the SBP treated and control groups. One possible explanation for this phenomenon might be the use of anesthesia throughout the experiment, which may have affected autonomic sympathetic nervous activity and diminished blood vessel capacity. Insulin resistance is also considered to be a significant pathological characteristic of MS and it has previously been reported that SBP improved insulin resistance as evaluated via glucose tolerance testing (GTT) and insulin dose-response curves (Yanping et al., 2013). Although the underlying mechanisms remain to be verified, our results suggest that SBP improves metabolic disorders and other complications of MS primarily through pathways inhibiting inflammation, improving lipid metabolism and anti-oxidative mitochondrial protection. In conclusion, this study demonstrated that SBP can improve glucose and lipid metabolism in a rat model of MS. SBP is a naturally formulated compound with minimal known side effects and shows great potential as a promising medicine for effective MS management. Post-treatment studies evaluating SBP are necessary for promoting entry of this compound into clinical guidelines.

## Author Contributions

LuS contributed to the conception and design of the study. DW, NZ, LZ, LiS, and LW performed the experiments and analyzed the data. DW and LuS wrote the manuscript. All authors contributed to manuscript revision and approved the submitted version.

## Conflict of Interest Statement

LW was employed by company Hutchison Pharmaceuticals, Shanghai, China. LiS was employed by company Wanleibio Co., Ltd., Shenyang, China. All other authors declare no competing interests.
